# Better Safe Than Sorry: A Rare Case of a Laryngeal Foreign Body and the Unconventional Use of Cook® Airway

**DOI:** 10.7759/cureus.52918

**Published:** 2024-01-25

**Authors:** Muhammad Syafiq H Musa, Zhi Xiang Yeoh, Mawaddah Azman

**Affiliations:** 1 Otolaryngology, National University of Malaysia, Kuala Lumpur, MYS; 2 Otorhinolaryngology, Hospital Sultanah Bahiyah, Alor Setar, MYS

**Keywords:** aspiration, pediatric, airway exchange catheter, cook airway, inhalation, foreign body larynx

## Abstract

Foreign body (FB) inhalation in the pediatric population is a common emergency referral in otolaryngology practice. Mismanagement can lead to significant morbidity or even mortality. Anesthesiologists conventionally use the Cook® airway exchange catheter (CAEC) during endotracheal tube exchange in the intensive care unit, but its usage as an oxygen conduit is beneficial in other airway procedures. A healthy two-year-old boy was brought to casualty for allegedly choking on a boneless chicken meat bolus during mealtime. The initial presentation showed that the child was comfortable with soft audible stridor without signs of respiratory distress. Bedside video laryngoscopy revealed a whitish FB in the proximity of the vocal cord. The patient was subjected to emergency direct laryngoscopy and bronchoscopy to retrieve the FB. Under general anesthesia, the true nature of FB was revealed, which was an embedded chicken bone into the laryngeal ventricle, causing a significant reduction of the rima glottis opening. CAEC was used to maintain oxygenation during the complex extraction process, and the child was discharged without any morbidity. Eyewitness history is an essential component in diagnosing FB inhalation in the pediatric population. Despite that, identifying potential difficulty is important to provide backup, especially in the case of unexpected events during managing airway emergencies.

## Introduction

Foreign body (FB) inhalation is a problem commonly encountered among infants. Mismanagement in the casualty can lead to significant morbidity or mortality. The curious nature and tendency for oral exploration of an infant puts them at a higher risk of accidental inhalation into the airway [1]. Despite close supervision by parents, FB inhalation still occurs, especially during meals. History remains the key element in raising suspicion of FB inhalation in children [1]. The majority of the inhaled FBs are located in the lower airway, in particular, the right and left bronchi. The laryngotracheal region is a rare site for FB dislodgement but can potentially lead to a life-threatening situation [2].

Emergency extraction of FB under general anesthesia is the cornerstone of management to avoid further disastrous sequelae to the child. Anesthesiologists primarily use the Cook® airway exchange catheter (CAEC) to act as a conduit to deliver oxygen during endotracheal tube (ETT) exchange in the intensive care unit [3].

Here, we report a rare case of FB aspiration in the larynx to highlight the misleading history that potentially complicates the removal of FBs and the non-conventional use of CAEC in the procedure.

## Case presentation

A healthy two-year-old boy was brought to the casualty by his father with a history of a choking incident during mealtime. The history gathered from his father suggested choking and vomiting episodes after taking a bolus of boneless chicken meat. The child was otherwise comfortable after a brief period of crying and noisy breathing. Clinically, he was afebrile and not dyspneic. A soft audible stridor was heard, but there was no respiratory distress. Intraoral examination and lung auscultation were unremarkable. The child was then subjected to flexible video laryngoscopy in the casualty and a glimpse of a whitish foreign material was seen in the proximity of the vocal cord.

**Figure 1:**
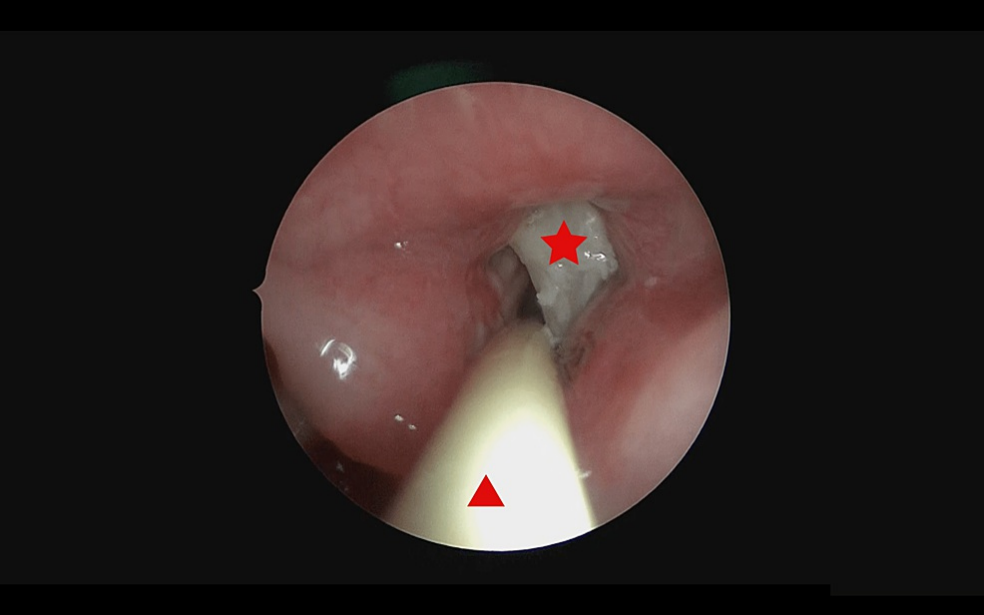
Endoscopic image of the foreign body embedded in the laryngeal ventricle (red star) with the Cook® airway placed in the rima glottis (red triangle).

**Figure 2:**
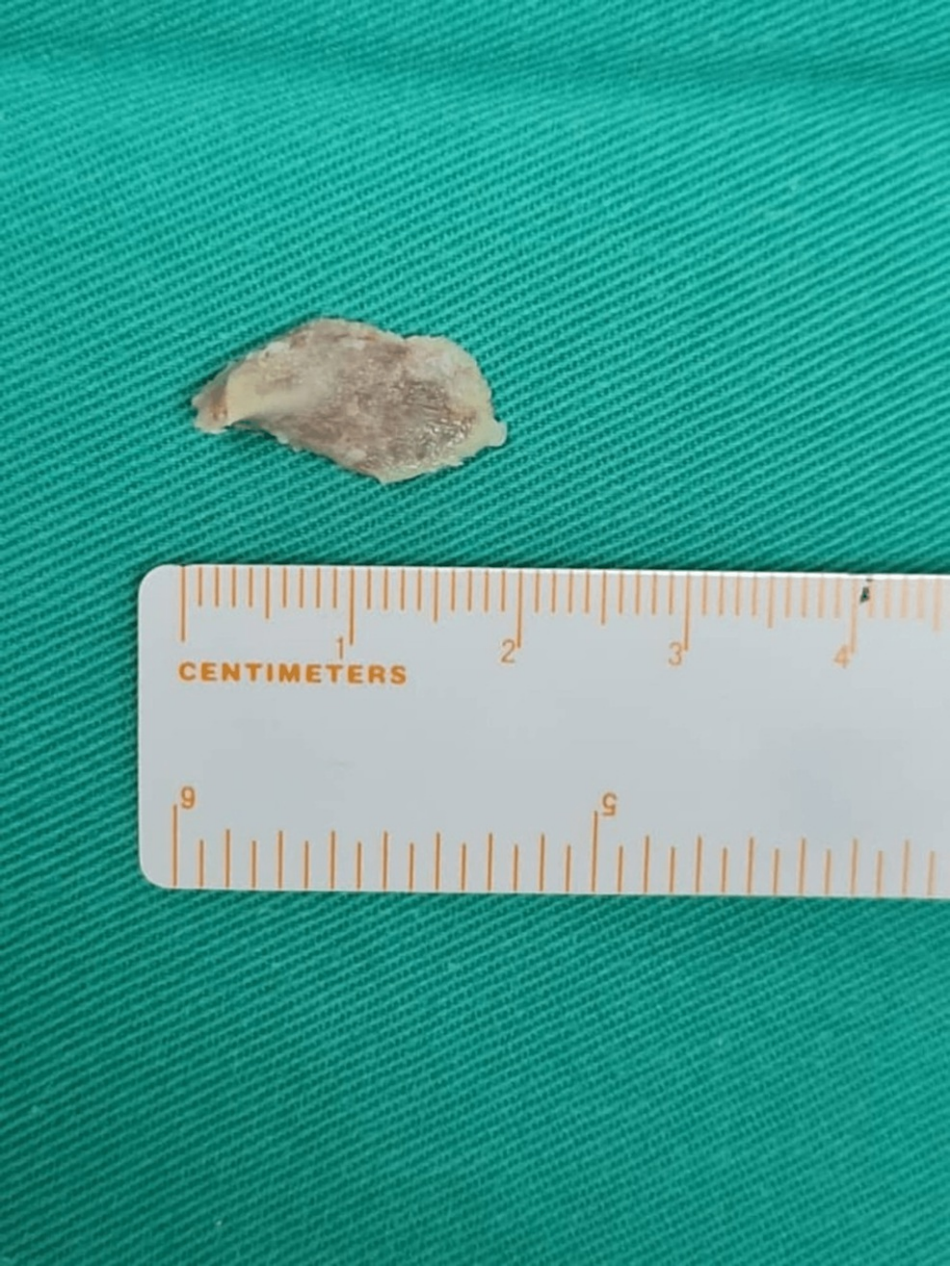
The removed foreign body (chicken bone) measuring 2 x 1 cm.

Emergency direct laryngoscopy and bronchoscopy were performed to retrieve the FB. Induction of anesthesia was achieved using intravenous propofol 20 mg (2 mg/kg) and remifentanil 20 µg (2 µg/kg). Suspension laryngoscopy examination revealed an obstructing FB oriented horizontally, embedded within the mucosa of the left ventricle (Figure 1). A quick decision was made to introduce CAEC into the limited available space at rima glottis for ventilation (Figure 1). The embedded FB was removed in one piece after a few manipulations in an attempt to dislodge it from the edematous mucosa using crocodile forceps (Figure 2). A rigid bronchoscope was then introduced to examine the lower airway to look for possible remaining FB. Upon completion, adrenaline and dexamethasone were packed momentarily over the previously lodged FB site.

The child was successfully extubated and was monitored in the pediatric high-dependency unit. He was comfortable under nasal prong oxygen supplementation after the procedure and was able to tolerate oral feeds. Flexible laryngoscopy before discharge showed minimal edema at the FB site with normal vocal cord mobility and patent airway. Repeated flexible endoscopy at the two-week follow-up showed resolution of edema and no active complaint from the parents.

## Discussion

FB inhalation is a common emergency referral received by the otorhinolaryngology team. The majority of patients are in the pediatric age group with peak incidence at the ages of 1 to 3 years [2,4-6]. Organic FB, particularly solid food items such as nuts and seeds, may be accidentally inhaled during meals, especially in infants [1]. Despite supervised feeding by an adult, accidents can occur due to the lack of crushing effect by the absence of molar teeth in infants which usually appear in the second year of life [1,7]. Boys are naturally more active than girls and are found to be more susceptible to FB inhalation [2,5,6,8].

Depending on the location of FB inhaled, patients come to the casualty with various presentations. History by eyewitnesses is the most essential component to lead the diagnosis, mainly in asymptomatic children [2]. Delayed diagnosis commonly occurs when a patient presents with late complications due to resolved symptoms after an acute inhalation event. Unexplained recurrent lung infections, chronic cough, and dyspnea are clues that raise a high index of suspicion of FB inhalation in children [2,9]. According to previous studies, the right main bronchus is statistically the most frequent site of FB dislodged, followed by the left main bronchus and laryngotracheal region [1,2,4-10].

Upper airway FB brings different challenges as it can present as sudden airway obstruction which is potentially life-threatening [6]. Some children also present hoarseness, aphonia, and wheezing depending on the degree of obstruction [6]. In cases of stable and cooperative children, a fibreoptic video laryngoscope aids the diagnosis with high accuracy [6]. A flexible video laryngoscope was used in our case and could identify the FB in the larynx. The child was later subjected to removal under general anesthesia. Despite the history confidently provided as boneless chicken meat by the parent, the attending physician should consider the possibility of different types of FBs as difficulty might be encountered during the extraction process. Proper planning of procedural steps and instruments required is important to allow a smooth procedure in the operation theater. Therefore, communication between surgeons, anesthetists, and operating staff is crucial to ensure safe surgery.

Rigid instruments, in particular rigid direct laryngoscope and bronchoscope, are important tools to the operating surgeon for the diagnosis and retrieval of FBs. CAEC is a small semi-rigid catheter primarily used by anesthetists during the exchange of ETT, especially in difficult airway cases [11]. It provides safe passage and can deliver sufficient oxygen during the entire process. Fayoux et al. presented an alternative use of CAEC in managing laryngotracheal stenosis in neonates and infants [12]. The incidence of hypoxemia was greatly reduced with a combination use of CAEC [11,12]. After FB was visualized using a laryngoscope in the anesthetized child’s larynx, we decided to introduce CAEC before the extraction to not only give extra minutes of oxygenation but also additional space for manipulation to remove the FB. It proved to be the best decision as the embedded FB was retrieved after a few attempts, without any immediate or delayed complications.

## Conclusions

Inhaled FB is a common problem in pediatrics and still presents different challenges to the surgeon. Prompt decisions on anesthetic techniques to be used intraoperatively and the ability to identify potential difficulty as well as unexpected risk are key elements to avoid lethal complications in the operation theater. Back-up plans should be made by surgeons and anesthetists in airway cases to avoid undesirable morbidity and mortality.

## References

[1] Jacobs IN, Jatana KR (2021). Current management of aerodigestive foreign bodies in children. Semin Pediatr Surg.

[2] Sultan TA, van As AB (2016). Review of tracheobronchial foreign body aspiration in the South African paediatric age group. J Thorac Dis.

[3] Pai Bh P, Shariat AN (2019). Revisiting a case of difficult airway with a rigid laryngoscope. BMJ Case Rep.

[4] Ngamsanga S, Vathanophas V, Ungkanont K, Tanphaichitr A, Wannarong T (2023). Pediatric respiratory tract foreign bodies in children: a systematic review. Auris Nasus Larynx.

[5] Kalyanappagol Kalyanappagol, Vijaykumar T, Kulkarni NH, Bidri LH (2007). Management of tracheobronchial foreign body aspirations in paediatric age group - a 10 year retrospective analysis. Indian J Anaesth.

[6] Passàli D, Lauriello M, Bellussi L, Passali G, Passali F, Gregori D (2010). Foreign body inhalation in children: an update. Acta Otorhinolaryngol Ital.

[7] Bellocchi G, Acquaviva G, Giammona Indaco F, Eibenstein A (2020). Foreign bodies in the pediatric age: the experience of an Italian tertiary care hospital. Acta Biomed.

[8] Gendeh BS, Gendeh HS, Purnima S, Comoretto RI, Gregori D, Gulati A (2019). Inhaled foreign body impaction: a review of literature in Malaysian children. Indian J Pediatr.

[9] Erol MK, Günendi T, Kaya F, Dörterler ME (2022). Foreign body aspiration in children: review of 198 cases from anesthesiology perspective. Harran Üniv Tıp Fakültesi Derg.

[10] Bukhari DH, Kabli AF, Alharthi TS, Sendi E, Rashed AA (2022). A rare case of a vocal cord foreign body in an infant: a case report. Cureus.

[11] Mort TC (2007). Continuous airway access for the difficult extubation: the efficacy of the airway exchange catheter. Anesth Analg.

[12] Fayoux P, Marciniak B, Engelhardt T (2009). Airway exchange catheters use in the airway management of neonates and infants undergoing surgical treatment of laryngeal stenosis. Pediatr Crit Care Med.

